# Rhegmatogenous Retinal Detachment in Anterior Scleritis With Ulcerative Colitis

**DOI:** 10.7759/cureus.61819

**Published:** 2024-06-06

**Authors:** Bannu Jayallan, Mohd Hasif Mustafa, Norshamsiah Md Din, Mae-Lynn Catherine Bastion

**Affiliations:** 1 Department of Ophthalmology, Hospital Canselor Tuanku Muhriz Universiti Kebangsaan Malaysia, Kuala Lumpur, MYS

**Keywords:** steroids, scleral buckle, ulcerative colitis, anterior scleritis, rhegmatogenous retinal detachment

## Abstract

Rhegmatogenous retinal detachment (RRD) is an ocular emergency as it is sight-threatening and requires urgent surgical intervention. Ulcerative colitis (UC) is an immune-mediated inflammatory bowel disease that can present with ocular manifestations. The objective of this case report is to share the rare presentation of RRD associated with UC leading to diagnosis and management dilemmas.

A 35-year-old man with active UC presented with a right chronic red eye for two months. The best corrected visual acuity (BCVA) was 6/6 in both eyes (OU). On examination, sectoral inferotemporal anterior scleritis (AS) with subclinical inferior RRD with peripheral holes in the lattice at the 6 o’clock position was noted. There was no posterior vitreous detachment. Optical coherence tomography (OCT) delineated the RRD objectively and was non-progressive for nine months. Barricade laser was given, in addition to intravenous methylprednisolone (IVMP), followed by a tapering dose of oral prednisolone and topical dexamethasone 0.1% over three months. Over a year, the scleritis resolved. However, six months later, while still on immunomodulating agents, the inferior RRD progressed on OCT. Segmental scleral buckle, indirect laser retinopexy, and subtenon triamcinolone injection were performed. IVMP 1 g per day was given for three days prior to surgery. Two months later, his BCVA was 6/6, with signs of fluid resorption and normal intraocular pressure. No recurrent AS was seen.

Treatment of non-progressive, subclinical RRD patients with UC and active AS can be delayed with regular follow-up. When RRD progressed and there was no AS activity, it was the window of opportunity for the success of scleral buckle and perioperative steroids.

## Introduction

Rhegmatogenous retinal detachment (RRD) is an ocular emergency as it is sight-threatening and requires urgent surgical intervention, especially in cases that threaten the macula. This intervention typically involves an external approach, such as scleral buckling, or an internal approach, such as pars planar vitrectomy. Both these repair procedures involve incisions in the sclera. The former requires a buckle, which is typically sutured to the sclera. The latter involves incisions in the scleral ranging from 0.6 to 0.9 mm in size [1].

Ulcerative colitis (UC) is an immune-mediated inflammatory bowel disease that can present with extra-intestinal manifestations, such as episcleritis, scleritis, uveitis, and leukocytoclastic vasculitis [2]. During the active phase of UC, there is a risk of developing surgically induced necrotizing scleritis (SINS); hence, the sclera should not be disturbed [3]. Less commonly, UC in association with scleritis may develop exudative retinal detachment (RD) and unlikely RRD [4].

Hence, the presence of RRD in a patient with UC represents a diagnostic and treatment dilemma. The purpose of this case report is to share the management of one such case.

## Case presentation

**Figure 1:**
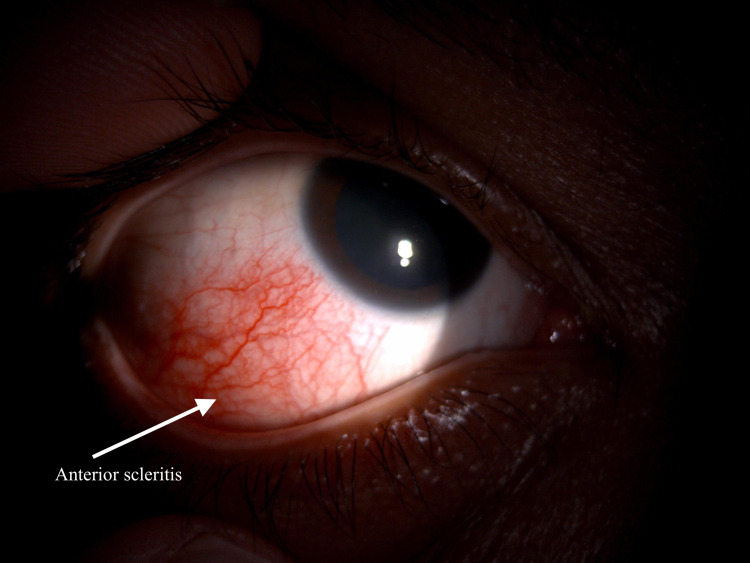
Anterior segment photo of the right eye showing a localized area of injection at the inferior temporal aspect. There is no blanching of the vessels seen when topical phenylephrine 2.5% is applied with mild discomfort suggesting anterior scleritis.

**Figure 2:**
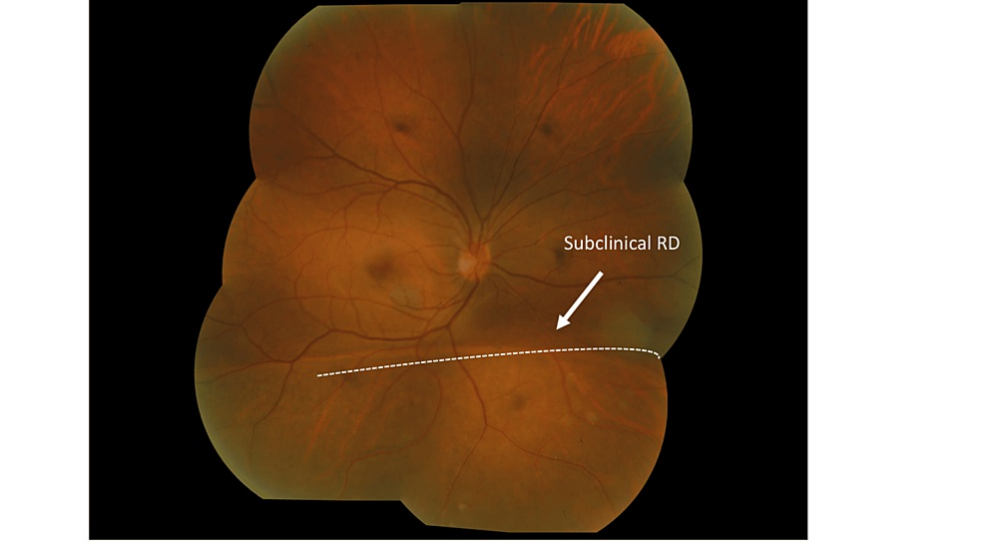
Dilated fundus photograph of the right eye showing demarcation line and subclinical RD (arrow) involving the inferior retina. The holes in the lattice are not visible due to their peripheral location. RD: retinal detachment.

A 35-year-old man with underlying active UC presented with right chronic eye redness for two months. There was mild eye discomfort with no blurring of vision. There was no pain, no eye discharge, no floaters or flashes of light, and no history of trauma to the eyes. The patient was given oral mesalazine and intravenous vedolizumab by his physician. On examination, his best corrected visual acuity (BCVA) was 6/6 in both eyes (OU). His refraction was -8.00D in the right eye (OD) and -7.00D in the left eye (OS), indicating high myopia. Anterior segment examination of the right eye revealed sectoral inferotemporal anterior scleritis (AS). It did not blanch with topical phenylephrine 2.5% (Figure 1). The cornea was clear. The anterior chamber was deep and quiet, the lens was clear, and intraocular pressure (IOP) was 11 mmHg OU. Dilated fundus examination showed subclinical inferior RD with peripheral holes in the lattice at the 6 o'clock position with no posterior vitreous detachment (Figure 2). The optic disc was pink with a 0.3-cup disc ratio. There was no tobacco dust seen in the anterior vitreous. There was no shifting fluid or proliferative vitreoretinopathy detected. No evidence of posterior scleritis in B-scan ultrasonography was noted. The left eye was unremarkable. The patient was treated as anterior scleritis with subclinical RD secondary to active UC.

**Figure 3:**
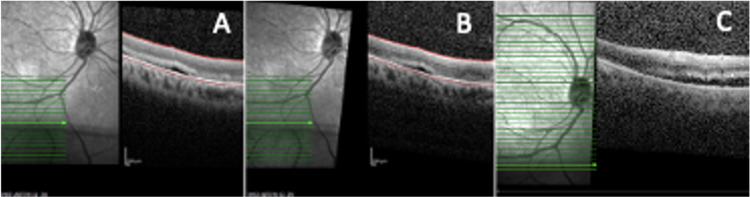
Serial optical coherence tomography of subclinical rhegmatogenous retinal detachment (RRD) at (A) baseline, (B) after six months (no change), and (C) after 18 months (progression seen).

**Figure 4:**
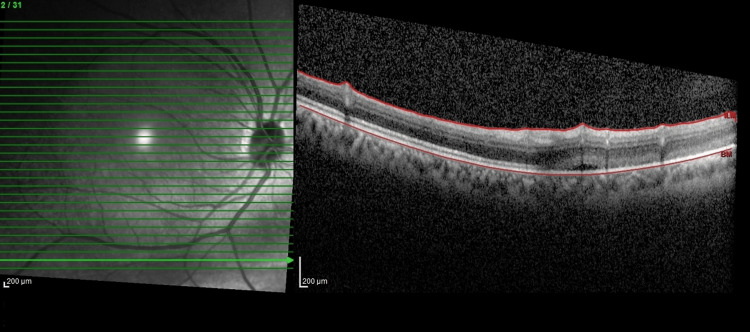
Optical coherence tomography postoperatively at one month shows resolving subretinal fluid and reattachment of the retina.

The patient was started on intravenous methylprednisolone (IVMP) 250 mg four times a day (QID) for three days, followed by a tapering dose of oral prednisolone 1 mg/kg and topical dexamethasone 0.1% over three months. Based on our clinical judgment, we anticipate conservative management may be inferior and not ideal in this patient despite subclinical RD not needing active management, hence a barricade laser was given to the RD. The patient also had a consultation with the physician and an evaluation of his systemic condition. Optical coherence tomography (OCT) of the inferior retina delineated the RD objectively from the time of this presentation, and it was non-progressive for nine months. Over a year, the scleritis resolved. However, six months later, while still on oral mesalazine 1 g per day and intravenous vedolizumab 300 mg every eight weeks for UC, the inferior RD progressed clinically and was evident by OCT (Figure 3). At this time, there was no active scleritis. The patient was treated as RRD. Scleral buckle band-40 and band-276 were soaked with gentamicin and then implanted 360 degrees, and indirect laser retinopexy and subtenon triamcinolone injection were performed under general anesthesia. The buckle was sutured to the sclera using Ethibond 5/0. The patient was given IVMP 1 g/day for three days before the procedures as an immunosuppressive agent. Postoperatively, the patient was prescribed topical ciprofloxacin (Ciloxan^TM^, FDC Pharmaceutical, Aurangabad, India) and dexamethasone (Maxidex^TM^, Alcon, Fort Worth, TX) two hourly for one week and then tapered to four hourly for three weeks then six hourly for two weeks and stopped. In addition to that, the patient was prescribed ointment dexamethasone, neomycin, and polymyxin B ointment (Maxitrol^TM^, Alcon, Fort Worth, TX) at night for six weeks. Two months later, his BCVA was 6/6, with signs of fluid resorption (Figure 4) and normal IOP. No recurrent anterior scleritis was seen.

## Discussion

RD is defined as the separation of the neurosensory retina from the retinal pigment epithelium (RPE), which leads to fluid accumulation within this potential subretinal space (SRS). RRD, in which a full-thickness defect occurs in the retina resulting in separation from the RPE, is a common cause of RD. The integrity of the retinal layer interface is maintained by regular adhesive forces, such as the mucopolysaccharide in the SRS, oncotic pressure, and metabolic ion transfer to and from the retinal pigmented epithelium. Insult to these factors allows the fluid into the potential SRS [1].

Subclinical RD is a term used when a patient with peripheral RD is asymptomatic. Typically in such patients, the subretinal fluid extends no more than one optic disc diameter away from the nearest retinal break and not more than two disc diameters posterior to the equator [5]. The inferior and subclinical nature of the RD in this patient permitted a monitoring period in the presence of a barricade laser. This monitoring period allows the patient to respond to immune therapy and to monitor the rate of progression of the RD given that an exudative component could not be excluded.

On the other hand, a solid vitreous interface may temporarily plug the retinal defect, thus preventing fluid entry into the SRS. This is common in younger individuals who have not undergone vitreous syneresis or posterior vitreous detachment [6]. This could have occurred in this patient, resulting in a stable RD for over one year. On the other hand, the possibility of exudative detachment corresponding to the location of the scleritis seen externally was entertained. Hence, a trial of pulse methylprednisolone was given. Interestingly, the RD remained stable and did not increase. Another risk factor that can cause RRD in this patient is the pre-existing holes in the lattice degeneration.

UC is a systemic inflammatory disease that predominantly affects the bowel. It can also affect other organs, including the eyes [2]. The etiology and pathophysiology remain unclear; however, there are a few theories that have been postulated, such as genetic susceptibility with higher racial predilection among Caucasians. Environmental factors such as diet and the use of antibiotics alter the gut microbes leading to the development of UC. Clinical manifestation is an immune response of the intestinal and extraintestinal organs to the autoantibody productions and the antigen-antibody complex circulating in the system [7]. These may deposit in the sclera and cause scleritis like in this patient. Treatment of UC is based on disease severity and response to treatment. In general, aminosalicylates such as mesalazine sulfasalazine and balsalazide are used as first-line therapy. Corticosteroids are the second-line therapy. Third-line therapy will be immunomodulators such as azathioprine and methotrexate. Biologics such as infliximab, adalimumab, and vedolizumab are used as fourth-line therapy [8]. Interestingly, ocular complications in UC can be due to the disease or medications used to treat the condition. Prolonged use of corticosteroid and biological drugs can lead to ocular morbidities such as cataracts, uveitis, optic neuropathy, open-angle glaucoma, and irritation to the cornea and eyelids [2].

The prevalence of ophthalmic manifestation, including AS, is 1.6%-5.4% in UC patients [9]. Lavric et al. reported that 28.1% of patients with posterior scleritis developed serous RD. About 40% of the patients had systemic autoimmune diseases such as UC [10]. Scleritis is often described as a boring or piercing pain. Interestingly, this patient only had mild discomfort. A large cohort study described 42.4% of patients with scleritis presented with mild pain [11]. The treatment of AS in UC includes steroids and cycloplegics to reduce inflammation and ocular pain [12]. IVMP 250 mg four times a day was prescribed in this patient and later converted to oral prednisolone and tapered slowly. Pulses of IVMP of 1 g/day followed by a tapering dose of oral prednisolone in patients with autoimmune disease have been shown to achieve better response [13,14]. Topical dexamethasone was also initiated and tapered slowly. This treatment regimen appears to have helped the AS in this patient to resolve. Interestingly the response was slow and required almost three months of therapy.

Monitoring of RRD progression has been made more objective by the usage of OCT. With peripheral OCT, the exact extent of the RRD can be documented. Using the progression function in the OCT machine, the amount of subretinal fluid and the margins of the RRD can be compared objectively. This was useful in this patient to monitor the progression of his RRD. It prompted intervention at the appropriate time when his scleritis had resolved.

The surgical options usually depend on the centers and the surgeon's preferences. The standard surgical options are scleral buckle, pars plana vitrectomy, and pneumatic retinopexy. Pneumatic retinopexy is not appropriate in this patient due to the inferior location of the tears and RD. Multiple studies compare the outcome of the scleral buckle and pars plana vitrectomy. There were no significant differences in the functional outcomes in one of the prospective randomized multicenter trials by Heimann et al. comparing scleral buckle and primary vitrectomy [15]. However, in a patient with autoimmune disease with a history of AS, vitrectomy port incisions are prone to inducing scleritis. Furthermore, the RRD in this patient was inferior, making gas tamponade for the retinal tear challenging. Cryotherapy in particular was avoided to reduce inflammation with a laser given to barricade the tear.

There were no particular guidelines on the ophthalmology follow-up for patients with UC. This patient, however, was monitored closely within six to eight weeks of interval. The scleritis was improving, while the RD remained stable. The treatment options for this patient had to be decided carefully as the risks of an early surgical intervention might have outweighed its benefits due to the risk of developing SINS, scleral melting, or perforation postoperatively in this patient. For instance, in a study, it was noted that 45% of patients with an underlying autoimmune disease developed more severe necrotizing scleritis compared to patients without an underlying autoimmune disease post surgery [16].

Cohen's case series showed no progression in 18 asymptomatic untreated RRD patients throughout 46 months of follow-up [17]. In a prospective cohort study by Byer, the incidence of progression of subclinical RD to clinical RD is less than 1% per year. Around 91% of the subjects had lattice degeneration with round atrophic holes similar to this patient. The author also concluded that subclinical RD should be reviewed regularly and opt for surgical treatment when it progresses to clinical RD [18]. However, we felt in this case the solid vitreous in this relatively young patient helped to delay the progression of the RD. It was fortunate that his scleritis resolved at the appropriate time.

Any ocular surgery, especially vitreoretinal surgeries, can trigger SINS. The risk of developing SINS is higher in active autoimmune disease. It is essential to assess and identify the high-risk patient before opting for surgery [3]. AS in this patient resolved with treatment, but the patient's RD progressed later, which was evident by OCT. The patient received an immunosuppressive dose of steroids three days before surgery to ensure his scleritis did not flare up during the postoperative period. Surgeons give steroids either before or after surgery in an attempt to reduce intraocular inflammation [19]. Drainage of the subretinal fluid externally was avoided to reduce incisions in the sclera. The patient subsequently underwent scleral buckling with an indirect laser retinopexy under general anesthesia. Triamcinolone is a glucocorticoid that is widely used in ophthalmology. Beardsley et al. reported treating posterior scleritis with subtenon triamcinolone was successful in their practice [14]. Johnson et al. said that the use of subtenon triamcinolone as an adjuvant treatment had improved inflammation in scleritis with a high relapse rate [20]. Subtenon triamcinolone was administered during this surgery to deliver periocular steroids for further postoperative control of inflammation in a patient with previous scleritis and known autoimmune disease. It was conveniently given during surgery and to the best of our knowledge, it has not been described before. This, combined with tapering doses of topical and oral steroids and his usual UC medications, seems to have prevented postoperative SINS in this patient.

## Conclusions

Treatment of non-progressive, subclinical RRD with underlying UC and active anterior scleritis can be delayed with regular follow-up. The anterior scleritis should be actively treated, and an exudative component should be considered. It is important to determine the type of retinal detachment (RRD or exudative RD) in cases like this. Clinicians should not assume that it is an exudative RD. A thorough indentation fundoscopy is mandatory in these patients. Monitoring for RD progression is more objective with serial OCT of the peripheral retina. When surgery is indicated in such cases, preoperative subtenon and postoperative steroids should be administered and tapered slowly. Minimal scleral disturbance can be achieved with successful repair of the RRD with a scleral buckle.

## References

[1] Feltgen N, Walter P (2014). Rhegmatogenous retinal detachment--an ophthalmologic emergency. Dtsch Arztebl Int.

[2] Troncoso LL, Biancardi AL, de Moraes HV Jr, Zaltman C (2017). Ophthalmic manifestations in patients with inflammatory bowel disease: a review. World J Gastroenterol.

[3] Ruiz-Lozano RE, Garza-Garza LA, Davila-Cavazos O, Foster CS, Rodriguez-Garcia A (2021). The clinical and pathogenic spectrum of surgically-induced scleral necrosis: a review. Surv Ophthalmol.

[4] Nakayama LF, Bergamo VC, Conti ML, Costa LA, Moraes NS, Ambrogini O Jr (2018). Frequency of ophthalmological posterior segment findings in patients with inflammatory bowel disease. Arq Gastroenterol.

[5] Colucciello M (2009). Rhegmatogenous retinal detachment. Phys Sportsmed.

[6] Yadav I, Purohit SD, Singh H (2021). Vitreous substitutes: an overview of the properties, importance, and development. J Biomed Mater Res B Appl Biomater.

[7] Silva FA, Rodrigues BL, Ayrizono ML, Leal RF (2016). The immunological basis of inflammatory bowel disease. Gastroenterol Res Pract.

[8] Blonski W, Buchner AM, Lichtenstein GR (2014). Treatment of ulcerative colitis. Curr Opin Gastroenterol.

[9] Lanna CC, Ferrari Mde L, Rocha SL, Nascimento E, de Carvalho MA, da Cunha AS (2008). A cross-sectional study of 130 Brazilian patients with Crohn's disease and ulcerative colitis: analysis of articular and ophthalmologic manifestations. Clin Rheumatol.

[10] Lavric A, Gonzalez-Lopez JJ, Majumder PD, Bansal N, Biswas J, Pavesio C, Agrawal R (2016). Posterior scleritis: analysis of epidemiology, clinical factors, and risk of recurrence in a cohort of 114 patients. Ocul Immunol Inflamm.

[11] Sainz de la Maza M, Molina N, Gonzalez-Gonzalez LA, Doctor PP, Tauber J, Foster CS (2012). Clinical characteristics of a large cohort of patients with scleritis and episcleritis. Ophthalmology.

[12] Harbord M, Annese V, Vavricka SR (2016). The first European evidence-based consensus on extra-intestinal manifestations in inflammatory bowel disease. J Crohns Colitis.

[13] Artaechevarria Artieda J, Estébanez-Corrales N, Sánchez-Pernaute O, Alejandre-Alba N (2020). Peripheral ulcerative keratitis in a patient with bilateral scleritis: medical and surgical management. Case Rep Ophthalmol.

[14] Beardsley RM, Suhler EB, Rosenbaum JT, Lin P (2013). Pharmacotherapy of scleritis: current paradigms and future directions. Expert Opin Pharmacother.

[15] Heimann H, Bartz-Schmidt KU, Bornfeld N, Weiss C, Hilgers RD, Foerster MH (2007). Scleral buckling versus primary vitrectomy in rhegmatogenous retinal detachment: a prospective randomized multicenter clinical study. Ophthalmology.

[16] Díaz-Valle D, Benítez del Castillo JM, Castillo A, Sayagués O, Bañares A, García-Sánchez J (1998). Immunologic and clinical evaluation of postsurgical necrotizing sclerocorneal ulceration. Cornea.

[17] Cohen SM (2005). Natural history of asymptomatic clinical retinal detachments. Am J Ophthalmol.

[18] Byer NE (2001). Subclinical retinal detachment resulting from asymptomatic retinal breaks: prognosis for progression and regression. Ophthalmology.

[19] Wei Y, Wang N, Chen F, Wang H, Bi C, Zu Z, Yang X (2014). Vitrectomy combined with periocular/intravitreal injection of steroids for rhegmatogenous retinal detachment associated with choroidal detachment. Retina.

[20] Johnson KS, Chu DS (2010). Evaluation of sub-tenon triamcinolone acetonide injections in the treatment of scleritis. Am J Ophthalmol.

